# MediNet: simplifying federated and privacy-preserving AI deployment in healthcare

**DOI:** 10.1093/bioinformatics/btag742

**Published:** 2026-10-06

**Authors:** Ramon Mateo-Navarro, David Sarrat-González, Dolors Pelegrí-Sisó, Xavier Escribà-Montagut, Fernando Sánchez-Lasheras, Juan R González

**Affiliations:** Bioinformatic Research Group in Epidemiology (BRGE), Barcelona Institute for Global Health (ISGlobal), Barcelona, 08003, Spain; Bioinformatic Research Group in Epidemiology (BRGE), Barcelona Institute for Global Health (ISGlobal), Barcelona, 08003, Spain; Bioinformatic Research Group in Epidemiology (BRGE), Barcelona Institute for Global Health (ISGlobal), Barcelona, 08003, Spain; BigOmics Analytics SA; Department of Mathematics, Faculty of Sciences, University of Oviedo, Oviedo, 33007, Spain; Institute of Space Sciences and Technologies of Asturias. (ICTEA), University of Oviedo, Oviedo, 33004, Spain; Bioinformatic Research Group in Epidemiology (BRGE), Barcelona Institute for Global Health (ISGlobal), Barcelona, 08003, Spain; CIBER en Epidemiología y Salud Pública (CIBERESP), Instituto de Salud Carlos III, Madrid, 28029, Spain

## Abstract

**Motivation:**

The application of Artificial Intelligence and Deep Learning (DL) in healthcare is increasingly feasible in principle, yet remains inaccessible in practice for most clinical institutions. Beyond the well-known challenge of data privacy regulations that prevent centralization of patient records, two further barriers limit adoption. First, deploying a federated learning infrastructure requires substantial technical expertise: configuring distributed training environments, implementing privacy-preserving mechanisms such as Differential Privacy (DP), and managing secure inter-institutional communication demands specialized knowledge that most healthcare organizations do not possess. Second, designing and configuring DL models (selecting architectures, tuning hyperparameters, interpreting results) currently requires machine learning expertise that clinical professionals, including researchers and physicians, typically lack. Existing federated learning frameworks address the infrastructure problem but impose a steep learning curve that effectively excludes non-specialized users. A solution that abstracts this complexity, enabling clinicians and biomedical researchers to design, launch, and monitor federated training processes without programming or machine learning expertise, remains absent from the field.

**Results:**

To address existing limitations, we introduce MediNet, a comprehensive server–client Federated learning (FL) platform that allows hospitals and research centers to train Machine Learning (ML) and DL models without moving or exposing sensitive data. It offers an intuitive web-based environment that simplifies FL, enabling users to easily select the model and define training criteria while administrators manage permissions and datasets, ensuring granular and autonomous data control. The system automatically constructs and generates the underlying technical configuration (e.g. Python/FL scripts) according to the parameters specified in the GUI. It then orchestrates the secure federated rounds and provides real-time monitoring, fully encapsulating the complexity of deployment and security. MediNet is built upon PyTorch as its primary framework, chosen for its robustness in DL model development along with its Differential Privacy (DP) libraries. Flower AI (Beutel *et al.* 2020) is used as the federated orchestration system. These underlying technologies are internal pillars of MediNet, essential for ensuring robustness, functionality, and strict adherence to privacy principles.

**Availability and implementations:**

The general information page for the MediNet software is available at https://isglobal-brge.github.io/MediNet. The MediNet software and its complementary tools are fully available under the MIT license on GitHub. The MediNetHub and MediNetNode code can be found on https://github.com/isglobal-brge/MediNetHub and https://github.com/isglobal-brge/MediNetNode.

## 1 Introduction

Artificial Intelligence (AI) has transformed numerous sectors, including healthcare. However, access to medical data remains heavily restricted due to privacy regulations such as General Data Protection Regulation (GDPR). This fragmentation of information across hospitals, combined with legal and security barriers that hinder the centralization of medical data creates significant obstacles for collaborative research. Also, limited AI expertise within many healthcare institutions presents significant challenges for the advancement of this technology in the health sector, and its adoption remains challenging (Lambert *et al.* 2023).

While established FL solutions are available, such as Flower (Beutel *et al.* 2020), FATE (Liu *et al.* 2021), SUBSTRA (Galtier and Marini 2019), or DataSHIELD (Gaye *et al.* 2014), technical complexity often limits their clinical adoption. These platforms frequently impose barriers, requiring specialized knowledge in cluster orchestration and command-line interfaces (e.g. FATE), or demanding that users write or modify Python code for the underlying local algorithms (e.g. SUBSTRA). Furthermore, DataSHIELD’s primary focus on R environments means its support for Python-based Deep Learning (DL) models is limited, restricting its use to statistical modelling mainly in epidemiological studies (Krogh *et al.* 2026). More broadly, none of these frameworks provides an end-to-end clinical workflow with a graphical interface designed for non-technical users, nor integrates dataset-level privacy budget management as a first-class feature. To address these limitations, MediNet offers a platform that abstracts the underlying FL infrastructure behind an accessible interface, enabling clinicians and biomedical researchers to design, launch, and monitor federated training processes without programming expertise, while natively integrating differential privacy (DP) budget management at the dataset level.

MediNet is particularly valuable in hospital environments, where patient information confidentiality is an absolute priority. In this context, MediNet facilitates the use of FL, enabling the training of DP and machine learning (ML) models without the need to share sensitive information between different healthcare institutions. To understand how MediNet materializes this decentralized approach, it’s crucial to examine its underlying architecture and its components.

## 2 Materials and methods

### 2.1 Implementation and architecture

The architecture of MediNet is based on a strategic selection of modern, well-established technologies and frameworks that provide a robust foundation for its core functionalities. The backend was developed in Python, using the Django framework to power the REST API and server logic. The frontend is implemented in JavaScript with modern UI components, ensuring a smooth and user-friendly experience. For the distributed learning component, the platform leverages the Flower AI framework, which simplifies the implementation of federated strategies. Deep learning tasks are handled using PyTorch, and an additional layer of security is incorporated through DP optimizers, which are implemented via the Opacus library during the training phase. This combination of technologies not only enhances MediNet’s internal capabilities but also facilitates its seamless integration with existing hospital infrastructures, a crucial aspect for its practical adoption.

This architecture materializes as a server-client platform where the MediNet server (hub) orchestrates the federated process (see Fig. 1). The MediNetHub orchestrates the process by sending specific commands to the APIs of the hospital’s MediNet Nodes: ping to verify availability, get_data to retrieve metadata and lists for authorized datasets only, and train to initiate FL rounds. Within each Server, the Flower AI framework manages communication with the local database and executes the training logic on the data, ensuring that the sensitive patient information never leaves the healthcare facility. The communication protocol between MediNetHub and each MediNetNode follows a structured five-step sequence. First, a discovery phase verifies node availability and authenticates the connection via an API key issued by the local Node Administrator. Second, a metadata retrieval step queries the node’s authorized dataset list and anonymous summary statistics (such as sample counts and feature distributions) without exposing individual records. Third, the Hub transmits a training configuration as a structured JSON payload, encoding the model architecture, hyperparameters, and, when enabled, differential privacy parameters including the noise multiplier (*σ*) and the maximum gradient clipping norm (C). Fourth, the Node executes local training using the Flower client on its private data, applying Opacus DP optimizers where configured. Fifth, only the updated model parameters (weights) are returned to the Hub for federated aggregation; raw clinical data never traverse the network boundary.

**Figure 1:**
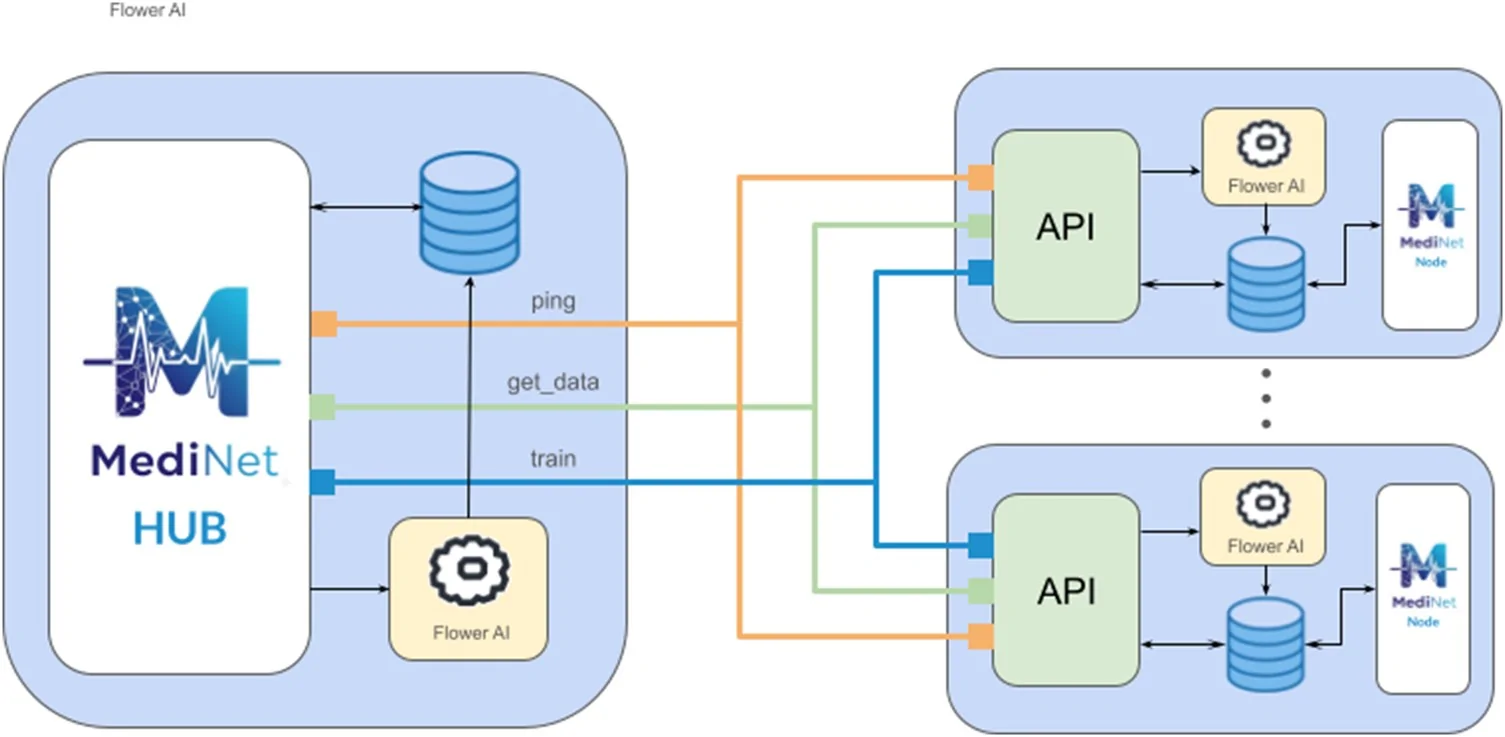
Architecture of MediNet server-client.

A fundamental security principle of MediNet’s design is that the Hub is treated as an untrusted orchestrator. Each MediNetNode independently validates every incoming request against its locally administered permission set, regardless of the Hub’s identity or claimed authorization. This zero-trust architecture (Rose *et al.* 2020) ensures that even if the Hub were compromised, a malicious actor could not exceed the permissions explicitly granted by the local Node Administrator, nor access datasets beyond those authorized for a specific researcher and training task. Budget accounting and data access controls are enforced exclusively at the Node, and the Hub has no mechanism to override or query them directly.

### 2.2 Integration with other systems

MediNet follows the principle of “sending the analysis to the data and returning only the aggregated results”, a core concept in federated analytics that ensures sensitive information remains protected. This approach facilitates seamless integration with existing hospital infrastructures through several key components: adapters for clinical data-management systems, a secure API for inter-component communication, and a modular design that supports environment-specific extensions for each hospital. This robust integration capability is complemented by an intuitive and comprehensive feature set, designed to empower healthcare professionals in the configuration, management, and monitoring of their AI models.

### 2.3 Core functionality

MediNet’s Hub core functionality is designed to empower users and streamline the FL workflow. Model configuration is managed through an intuitive Graphical User Interface (GUI) within the ‘Model Designer’ module, which abstracts the need for direct code manipulation. Users can visually construct neural network architectures by interacting with graphical elements, and each selection and parameter adjustment is automatically translated into a structured JSON representation. This JSON blueprint serves as a standardized input for the training process, enabling the definition of complex architectures without requiring explicit knowledge of programming languages. This seamless design is supported by the Automatic Model Builder, a component that transforms these JSON configurations into executable deep learning models, thereby automating the underlying framework-specific code generation and instantiation. Furthermore, MediNet supports diverse federated aggregation strategies and is built with a modular architecture that facilitates the seamless incorporation of new algorithms. The platform also includes a dedicated real-time monitoring dashboard for interactive visualization of training progress and key performance indicators, alongside a Model Comparison dashboard that allows for the evaluation of multiple trained models. Finally, Integrated Privacy Management is a core tenet of the system, employing DP techniques to robustly safeguard sensitive participant data throughout the entire FL lifecycle.

The core functionality of the MediNet Node has been designed to optimize the access and management of clinical data. To achieve this, the platform’s architecture is structured around three distinct user roles, each with a specific set of permissions that governs their interaction with the system. The Administrator role holds primary control over the local MediNetNode. They are solely responsible for the ingestion and management of the datasets hosted exclusively within their institutional Node, the creation of user accounts, and the administration of access permissions within their institution. Each Node operates as a sovereign data custodian: external actors (including the MediNetHub) interact exclusively through the Node’s authenticated REST API, and no operation can be executed without prior explicit authorization from the local Administrator. This design ensures that data governance remains entirely within the hands of the institution that owns the data, consistent with data sovereignty principles relevant to sensitive health data. The Administrator can grant or revoke per-dataset, per-researcher training permissions at any time. When a user account is created, the system can automatically generate a random password and a unique API key with a configurable validity period (default: 30 days), ensuring that access is time-bounded and regularly reviewed.

The Researcher role has been designed for programmatic interaction with the platform, serving as the main interface for designing and orchestrating Machine Learning and Deep Learning models. Through the API, users with this role can access the datasets’ metadata, which allows them to infer their structure and composition without directly accessing the sensitive information. This capability enables them to select and launch federated training processes, instantiating models on the pertinent datasets.

For the execution of these processes, it is a requirement for the Researcher to have active training permissions, which are administered by the Administrator role. The absence of this permission prevents the launching of training tasks, ensuring that control over the analysis workflows resides exclusively with the Administrators of each center. This architectural design reinforces the principle of “sending the analysis to the data and not the data to the analysis” a fundamental premise of MediNet.

Finally, the Auditor is a specialized role whose main function is to ensure the integrity and regulatory compliance of the platform. Unlike other roles, the Auditor has access to a completely different panel that allows them to perform exhaustive audits. This role can access the entire log system, visualize all uploaded datasets (including the data itself), and all activity performed by users on the platform. This allows them to validate that security protocols are followed and that no regulations are violated, thus fulfilling the task of conducting rigorous audits.

A key component of the system is the automated management of metadata. Whenever a new dataset is uploaded, the system automatically generates an associated metadata file. This file contains valuable information and anonymous metrics about the dataset, providing researchers with a comprehensive understanding of its composition before they begin their training processes.

### 2.4 Differential privacy budget management

When DP training is executed, the privacy guarantee is quantified through the (*ε*, *δ*)-differential privacy framework (Dwork and Roth 2014), where *ε* bounds the maximum information leakage per individual across all training rounds. Since *ε* is a finite and irrecoverable resource (each training round consumes a portion of the total budget), MediNet implements a budget accounting system managed exclusively at the Node level.

Each dataset hosted on a MediNetNode carries an associated privacy policy, configured by the local Administrator at upload time, which specifies: (i) the total *ε* budget allocated to that dataset across its lifetime, (ii) the maximum *ε* permitted per individual training job, and (iii) a sensitivity tier (low, medium, high) that guides default parameter recommendations. When a training job is submitted, the Node verifies that the requested configuration does not exceed the per-job budget before initiating any computation. Upon completion, the consumed *ε* is deducted from the dataset’s remaining budget, and any subsequent job that would exhaust the total budget is rejected. Budget state is stored locally and is inaccessible to the Hub. Recommended *ε* thresholds are informed by established literature: values below 1 offer strong formal guarantees, values in the range [1, 10] have been shown to yield useful model accuracy in practice (Abadi *et al.* 2016), and values above 10 provide limited privacy protection and are discouraged for sensitive health data (Jayaraman and Evans 2019).

A key practical requirement is the ability to estimate the *ε* cost of a training job before it is executed, so that both the Administrator and the Researcher can make informed decisions without inadvertently exhausting the budget. MediNet computes this estimate at job submission time using the Rényi Differential Privacy (RDP) accountant (Mironov 2017), as implemented in the Opacus library. Given a job configuration specifying the noise multiplier *σ*, the local batch size *b*, the local dataset size *N*, the number of local epochs *e*, and the number of federated rounds *R*, the total number of gradient steps is computed as *T* = *e* × ⌈*N*/*b*⌉ × *R*, where *e* × ⌈*N*/*b*⌉ follows the standard DP-SGD formulation (Abadi *et al.* 2016) and the factor R extends this to the federated setting. The per-step subsampling rate is *q* = *b*/*N* (Abadi *et al.* 2016). The RDP accountant then computes the privacy cost accumulated over *T* steps at subsampling rate *q* with noise multiplier *σ* (Mironov *et al*. 2019), and converts the result to the (*ε*, *δ*)-DP guarantee for a fixed target *δ* (commonly set to 1/*N* in practice). This pre-flight estimate is presented to the Researcher prior to job launch and is also used by the Node to verify that the projected *ε* does not exceed the per-job budget limit. The actual *ε* is recomputed post-training by Opacus and recorded as the authoritative consumed value. It is important to note that the consumed *ε* increases with the number of rounds *R* and epochs *e*, and decreases with larger noise multipliers *σ* (a relationship that the interface surfaces via the literature-based recommendations described above, guiding users towards configurations that balance model utility and privacy cost).

A persistent challenge in privacy-preserving model development is the tension between the iterative nature of model design and the finite privacy budget. Designing an effective DL architecture requires multiple training iterations to tune hyperparameters, assess convergence, and validate preprocessing choices (all of which, if run against the production dataset, would cumulatively erode the available *ε*). To address this, MediNet introduces the concept of an experimental dataset split. At upload time, the Node Administrator may optionally reserve a small subset of the dataset (configurable between 10% and 50% of the total samples) as an experimental partition. This split is performed as a physical separation of records at ingest time, resulting in two disjoint subsets: the experimental subset and the production dataset. Training runs executed against the experimental subset carry no *ε* accounting and do not affect the production budget, allowing the Researcher to iterate freely on model architecture and hyperparameters. Once a stable configuration is identified, the definitive training is launched against the production dataset under strict budget enforcement. This approach mirrors established machine learning practices of reserving a development subset (here motivated not by generalization concerns but by the finite and irrecoverable nature of the privacy budget). The experimental split is entirely optional; Administrators who prefer to manage budget allocation manually may choose not to configure it.

Finally, MediNet recognizes that the circumstances under which a dataset is used may evolve over time (for instance, when a new research project begins under a fresh ethics approval, or when additional patient consent has been obtained that justifies renewed data usage). To accommodate these scenarios, the Node Administrator may approve a budget reset request, restoring the dataset’s *ε* budget to its original configured value. Such requests are initiated by the Researcher and require explicit Administrator approval before taking effect. This operation is logged immutably in the audit trail, recording the timestamp, the identity of the Administrator who performed the reset, the previous budget state, and the justification provided. Budget resets are intentionally restricted to the Administrator role and require explicit confirmation, ensuring that no accidental or unauthorized erasure of privacy accounting can occur.

## 3 Practical use case and conclusions

### 3.1 Practical use case

Prior to any federated training, the Node Administrator performs the initial platform configuration. In our use case, the Administrator uploaded the Heart Attack Prediction dataset to the MediNetNode instance and configured its associated privacy policy, setting a total *ε* budget of 15.0 and a per-job maximum of 4.5, consistent with privacy budgets shown to maintain useful model accuracy in clinical ML applications (Abadi *et al.* 2016). A 20% experimental partition (1752 records) was optionally configured to allow the Researcher to iterate on model design without consuming the privacy budget; the privacy-accountable production run subsequently trains on the full dataset. The Administrator then created a Researcher account, granted training permissions on the uploaded dataset, and issued an API key with a 30-day validity period.

To evaluate MediNet’s capability for fast and iterative DL model optimization in a healthcare context (a detailed description is available at https://isglobal-brge.github.io/MediNet/use-cases.html), we utilized a synthetic version of the public Heart Attack Prediction dataset composed of 8762 patient records and 25 clinical features as input, of which a 20% experimental partition (1752 records) was configured. After pre-processing and transformation (e.g. One-Hot Encoding of categorical strings), the input dimensionality was increased to 52 features for the DL model, with the objective of predicting the binary variable Heart attack risk. To simulate a distributed hospital environment, the dataset was hosted on a remote server where MediNetNode was installed and configured with authenticated access.

It is important to note that the MediNet federated workflow represents a batch computation paradigm: training tasks are submitted as complete, pre-configured jobs and executed asynchronously, in contrast to REPL-based federated analytics platforms (such as DataSHIELD) which support interactive, query-driven data exploration. MediNet is designed specifically for model training and evaluation tasks; its dataset metadata-sharing capabilities offer a lightweight form of dataset introspection, but the platform does not aim to replicate interactive analytical workflows.

Assuming the role of the Researcher, we describe the steps required to operate the platform. For a detailed visualization of the MediNet deployment and execution workflow, please refer to the extended use case available on the MediNet website. We initiated the workflow (see Fig. 1 in the MediNet use-case documentation https://isglobal-brge.github.io/MediNet/versions/1.0/use-cases.html). We then logged into the MediNetHub web interface and provided the necessary credentials for the remote server connection (see Fig. 2 in the MediNet use-case documentation https://isglobal-brge.github.io/MediNet/versions/1.0/use-cases.html). Once the secure connection was established, we proceeded to the model design interface.

There, we designed a DL model using a sequential neural network (MLP). This architecture consisted of an input layer of 52 neurons, followed by two dense hidden layers of 64 and 32 neurons respectively, both with ReLU activation, and an output layer with Sigmoid activation for binary classification (see Fig. 4 in the MediNet use-case documentation https://isglobal-brge.github.io/MediNet/versions/1.0/use-cases.html). Training was initially configured with a Batch Size of 32 and 20 epochs, using the Adam optimizer and the Binary Cross-Entropy loss function.

To demonstrate MediNet’s hot-reconfiguration capability in a distributed setting, we rapidly re-evaluated the impact of Batch Size by modifying this hyperparameter to 16 via a simple update in the interface. MediNetHub managed the orchestration and re-distribution of the task to the remote server transparently and seamlessly, allowing the immediate launch of the new training without requiring any other parameter adjustments. Both experimental runs were executed under DP-SGD with the minimum noise multiplier enforced by the Node’s RDP accountant for the experimental partition (*σ*  =  0.957 for *b* = 32; *σ*  =  0.808 for *b* = 16), consuming a theoretical privacy cost of *ε*  ≈  4.49 and *ε*  ≈  4.46 respectively, without debiting the dataset’s lifetime budget.

Finally, a third production run was launched with an explicit noise multiplier of *σ*  =  0.71, which formally consumed *ε*  =  3.82 (*δ*  =  10^−^^5^) from the dataset’s privacy budget of *ε*  =  15.0. Results across all three runs are summarized in Table 1. Accuracy remained stable across configurations (∼0.65), while the DP production run incurred a moderate loss increase of +0.357 relative to the *b* = 32 experimental baseline. F1 scores showed higher variability across independent runs, which is attributable to the stochastic nature of DP-SGD combined with the class imbalance of the dataset. These results demonstrate the added value of MediNet in accelerating the iterative development of privacy-preserving clinical models: the platform executed three distinct training configurations end-to-end against a remote node while correctly enforcing and accounting for the differential-privacy budget *ε* = 3.82 debited from the dataset’s lifetime budget of 15.0 for the production run, with the experimental runs consuming none.

**Table 1:** Federated training results on the Heart Attack Prediction dataset (10 rounds, single client, 20 epochs).

Run	σ	Batch	Acc	Loss	FI
Experimental (*b *= 32)	0.957	32	0.655	1.595	0.028
Experimental (*b *= 16)	0.808	16	0.654	1.637	0.024
DP Production	0.710	32	0.650	1.959	0.011
DP cost vs. exp (*b* = 32)			−0.005	+0.357	−0.017

DP production run: *ε*  =  3.82, *δ*  =  10^−^^5^. Experimental runs consume no privacy budget.

Finally, the Auditor role provides an independent verification layer. In our scenario, the Auditor accessed the dedicated audit panel to review the complete activity log for the dataset: three training jobs had been executed (Batch Size 32, Batch Size 16, and the DP run), with timestamps, Researcher identity, model configuration, and (for the DP job) the *ε* consumed and remaining budget all recorded immutably. The Auditor confirmed that no training had been attempted beyond the permissions granted by the Administrator, that no dataset had been accessed outside authorized sessions, and that the privacy budget had been correctly decremented.

### 3.2 Conclusions and discussion

MediNet addresses the critical challenge that data privacy poses to AI in the healthcare sector. By adhering to the principle of bringing the analysis to the data rather than the reverse, it shares a foundational philosophy with federated analytics frameworks such as DataSHIELD. It is worth distinguishing, however, that MediNet operates as a batch model training system rather than an interactive analytics platform: its contribution lies in democratizing the development and deployment of ML and DL models in distributed clinical settings, complementing rather than replacing query-based federated analytics tools. In both cases, sensitive patient information never leaves the institution, ensuring confidentiality under frameworks such as GDPR and HIPAA. The platform further democratizes access to AI through its intuitive web interface and JSON-based model configuration, enabling healthcare professionals without advanced programming skills to design, train, and evaluate models. The always-active integration of DP optimizers, governed by a per-dataset budget accounting system managed exclusively at the Node level, reinforces this privacy commitment throughout the entire federated training lifecycle.

MediNet’s design, with its modular architecture, PyTorch compatibility, and features such as the automatic model builder and real-time monitoring, gives it considerable potential for adoption in research and clinical practice. These features simplify the iteration and development of effective models, significantly reducing the programming burden on researchers. With a forward-looking research agenda, the next stages of MediNet’s development will focus on incorporating new federated aggregation strategies and enhancing the tools to optimize the design of complex models. A key objective is to integrate the capability to map and transform heterogeneous databases to industry standards like OMOP (Stang *et al.* 2010), which will reduce the data preprocessing load and facilitate interoperability between centers. These lines of research aim to accelerate the development process and iteration to achieve increasingly robust and clinically relevant AI models. Ultimately, MediNet successfully establishes a viable, secure framework that addresses the critical need for collaborative AI development while strictly maintaining data ownership within the healthcare ecosystem.

## Data Availability

The original dataset is publicly available on Kaggle https://www.kaggle.com/datasets/iamsouravbanerjee/heart-attack-prediction-dataset. The cleaned and preprocessed dataset used in the proof-of-concept evaluation is available for download directly from the MediNet use-case webpage.
